# Natural Inhibitors Targeting the Localization of Lipoprotein System in *Vibrio parahaemolyticus*

**DOI:** 10.3390/ijms232214352

**Published:** 2022-11-18

**Authors:** Jiawen Liu, Jinrong Tong, Qian Wu, Jing Liu, Mengqi Yuan, Cuifang Tian, Huan Xu, Pradeep K. Malakar, Yingjie Pan, Yong Zhao, Zhaohuan Zhang

**Affiliations:** 1 College of Food Science and Technology, Shanghai Ocean University, 999# Hu Cheng Huan Road, Shanghai 201306, China; 2 Food Quality Supervision and Testing Center of the Ministry of Agriculture and Rural Affairs (Shanghai), Shanghai Center of Agri-Products Quality and Safety, Shanghai 201708, China; 3 Laboratory of Quality & Safety Risk Assessment for Aquatic Products on Storage and Preservation (Shanghai), Ministry of Agriculture and Rural Affairs, 999# Hu Cheng Huan Road, Shanghai 201306, China; 4 Shanghai Engineering Research Center of Aquatic-Product Processing & Preservation, 999# Hu Cheng Huan Road, Shanghai 201306, China

**Keywords:** natural inhibitors, the localization of lipoprotein system, LolB, *Vibrio parahaemolyticus*, virtual screening, molecular docking

## Abstract

The localization of lipoprotein (Lol) system is responsible for the transport of lipoproteins in the outer membrane (OM) of *Vibrio parahaemolyticus*. LolB catalyzes the last step in the Lol system, where lipoproteins are inserted into the OM. If the function of LolB is impeded, growth of *V. parahaemolyticus* is inhibited, due to lack of an intact OM barrier for protection against the external environment. Additionally, it becomes progressively harder to generate antimicrobial resistance (AMR). In this study, LolB was employed as the receptor for a high-throughput virtual screening from a natural compounds database. Compounds with higher glide score were selected for an inhibition assay against *V. parahaemolyticus*. It was found that procyanidin, stevioside, troxerutin and rutin had both exciting binding affinity with LolB in the micromolar range and preferable antibacterial activity in a concentration-dependent manner. The inhibition rates of 100 ppm were 87.89%, 86.2%, 91.39% and 83.71%, respectively. The bacteriostatic mechanisms of the four active compounds were explored further via fluorescence spectroscopy and molecular docking, illustrating that each molecule formed a stable complex with LolB via hydrogen bonds and pi–pi stacking interactions. Additionally, the critical sites for interaction with *V. parahaemolyticus* LolB, Tyr108 and Gln68, were also illustrated. This paper demonstrates the inhibition of LolB, thus, leading to antibacterial activity, and identifies LolB as a promising drug target for the first time. These compounds could be the basis for potential antibacterial agents against *V. parahaemolyticus*.

## 1. Introduction

*Vibrio parahaemolyticus* is an important foodborne pathogen widely distributed in aquatic products, and marine and estuarine environments [1]. It not only infects the *Penaeus vannamei* juvenile, leading to acute hepatopancreatic necrosis disease (AHPND) and serious economic losses [2,3], but also causes serious foodborne diseases, such as diarrhea and sepsis [4], which pose a great threat to human health and food safety. Meanwhile, the antimicrobial resistance (AMR) of bacteria, including *V. parahaemolyticus*, is severely increasing, aggravating the negative effect of bacteria on global health and the economy. If we do not suppress their development, drug-resistant superbugs will claim 10 million lives a year and cost the global economy a cumulative $100 trillion by 2050 [5].

As a Gram-negative bacterium, *V. parahaemolyticus* possesses an outer membrane (OM) as a solid barrier to resist the entry of detrimental factors, like antibiotics. Lipoprotein is an essential part of the OM, which is associated with the pathogenic mechanism [6]. It plays an important role in important physiological processes, including cell membrane biogenesis, adhesion and invasion, generation of drug resistance and so on [7]. In *V. parahaemolyticus* cells, the OM lipoprotein is synthesized and reaches the periplasmic leaflet of the inner membrane (IM), prior to transport to its correct location through the localization of the lipoprotein (Lol) system during OM biogenesis [8]. The Lol system is composed of five proteins, LolA-E [9]. Lipoproteins are pushed across the periplasm with the aid of chaperone LolA, from the ABC transporter LolCD_2_E complex anchored in the IM to the LolB embedded in the OM inner leaflet, powered by adenosine 5′-triphosphate (ATP) hydrolysis in the cytoplasm, and are incorporated into the OM by LolB [10].

Multiple novel sterilization techniques were developed to kill *V. parahaemolyticus*, such as ultraviolet light-emitting diodes [11], electrolyzed water [12] and photodynamic inactivation [13]. Under the background of increasingly severe AMR, a novel sterilization technology targeting OM biogenesis is an optional strategy [14]. A bactericidal agent targeting the Lol system is capable of blocking the transfer of the OM lipoprotein, disrupting the bacterial OM, and, eventually, eliminating *V. parahaemolyticus*. Inhibitors of LolCD_2_E and LolA, the inner membrane ABC transporter and the periplasmic chaperone in the Lol system, respectively, have already been reported [15]. Given that LolB protein is of great significance for an intact OM and the survival of *V. parahaemolyticus*, here, we, for the first time, report compounds targeting *V. parahaemolyticus* LolB by high-throughput virtual screening. The antibacterial activities and the mechanisms of interaction with LolB were also studied. These inhibitors of LolB provide a novel potential sterilization strategy for *V. parahaemolyticus*.

## 2. Results

### 2.1. Virtual Screening of Natural Compound Database

Virtual screening (VS) is the use of in silico techniques to screen active compounds, based on the compound database [16]. Using the molecular docking operation between compounds and drug targets, VS quickly selected active compounds with potential to be drugs from dozens to millions of molecules, greatly reducing the number of compounds for experimental screening, shortening the research cycle and decreasing the cost of drug development [17,18]. Therefore, VS has become one of the most promising tools for drug development.

**Figure 1:**
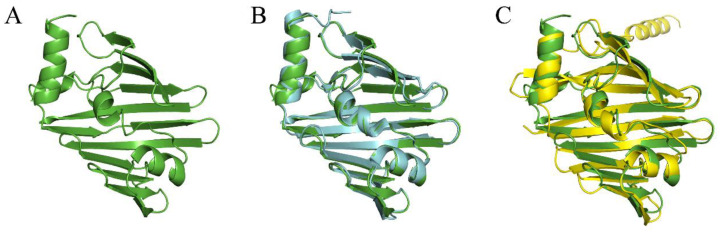
Homology model of *V. parahaemolyticus* LolB protein. (**A**): *V. parahaemolyticus* LolB (residues 36-211) modeled by Swiss-model. (**B**): Superposition of *V. parahaemolyticus* LolB model (green) with *E. coli* LolB crystal structure (cyan, PDB 1IWM). The rmsd was 0.374 over 137 C^α^. (**C**): Superposition of *V. parahaemolyticus* LolB model (green) with full-length structure of *V. parahaemolyticus* LolB (yellow) predicted by Alphafold. The rmsd was 1.033 over 141 C^α^.

The homology model of *V. parahaemolyticus* LolB protein used *Escherichia coli* (strain K12) LolB protein (PDB: 1iwm) [19] with 35% identity as template, where residues 36-211 of *V. parahaemolyticus* LolB were modeled successfully (Figure 1A). The model was aligned with *E. coli* LolB crystal structure and full-length *V. parahaemolyticus* LolB predicted by Alphafold, which showed high structural similarity (Figure 1B,C). In the established LolB model, as shown in the Ramachandran plot (Figure S1), 89.8% of residues were located in the completely conformation-allowed region, 10.2% in the conformation-allowed region, and no residue in conformational-disallowed region, indicating that these residues had good conformational space. The LolB model was of ideal quality for subsequent virtual screening.

Based on the screening of 39,000 molecules in the Cayman/2D structure database, 70 compounds that bind to the LolB protein were screened (Figure S2), and Table 1 lists the top 10 compounds with good glide scores to the target protein. With respect to the glide score, the lower score represents higher affinity with the receptor. The screened top 10 compounds had glide scores of −12.023~−7.781, lower than −6, demonstrating superior binding capability targeting *V. parahaemolyticus* LolB.

### 2.2. Inhibition Effect of the Top 10 Compounds on V. parahaemolyticus

**Figure 2:**
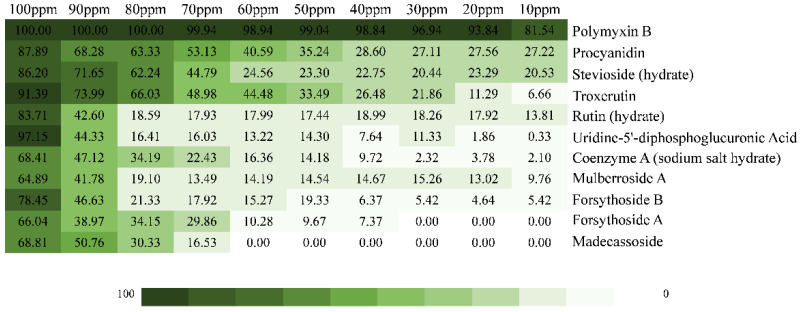
Inhibition effect of 100–10 ppm top 10 compounds on *V. parahaemolyticus*.

The listed top 10 compounds were applied to analyze their antibacterial effects on *V. parahaemolyticus* RIMD 2210633 by measuring the inhibition rates under a concentration gradient of 100–10 ppm (Figure 2). The results showed that these compounds had concentration-dependent bactericidal performances, among which procyanidin, stevioside, troxerutin and rutin presented relatively ideal inhibitory effects on *V. parahaemolyticus*, with inhibition rates of 100 ppm of 87.89%, 86.2%, 91.39% and 83.71%, respectively.

### 2.3. Fluorescence Spectroscopy of Active Compounds with LolB

LolB protein produces endogenous fluorescence under UV irradiation since it contains three aromatic amino acid residues, namely, tryptophan, tyrosine, and phenylalanine. When protein reacts with substances, the fluorescence intensity of fluorophore decreases, known as fluorescence quenching [20]. Therefore, fluorescence spectroscopy is widely applied for studying the interaction between proteins and inhibitors. In order to verify whether the reason why procyanidin, stevioside, troxerutin and rutin inhibited the activity of *V. parahaemolyticus* was the targeting effect on LolB protein, the fluorescence spectra of the interactions between the four active compounds and the isolated LolB protein were further measured, respectively.

**Figure 3:**
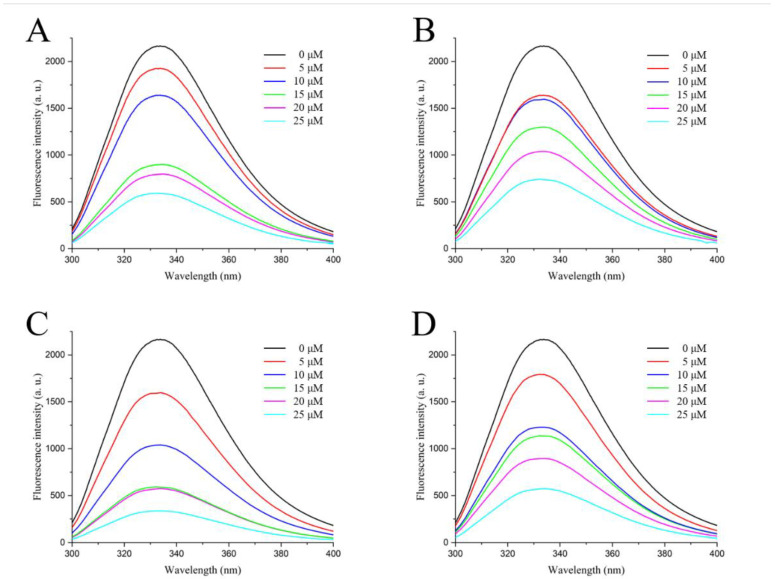
Fluorescence spectra of active compounds ((**A**): Procyanidin, (**B**): Stevioside, (**C**): Troxerutin, (**D**): Rutin) interacting with *V. parahaemolyticus* LolB protein.

As shown in Figure 3, the fluorescence emission peak emerged at 334 nm when the excitation wavelength was 280 nm, and the fluorescence intensity of LolB protein without quencher was 2166 a. u. The addition of procyanidin, stevioside, troxerutin and rutin significantly decreased the fluorescence intensity of LolB protein in a concentration-dependent manner from ~2000 a. u. at 0 μM to ~500 a. u. at 25 μM of active compounds. This suggested that the four molecules interacted with the LolB protein, resulting in fluorescence quenching of the LolB protein. Furthermore, with the increase of the concentration of active compounds, a blue shift of the fluorescence emission peak was observed in all fluorescence spectra (Figure 3), which implied that the interactions between LolB and the compounds possibly changed the microenvironment around LolB amino acid residues, so that the hydrophobicity of LolB increased and the polarity decreased. Together, fluorescence quenching and blue shift demonstrated that procyanidin, stevioside, troxerutin and rutin bound to LolB.

### 2.4. Fluorescence Quenching Mechanism of Active Compounds on LolB

**Figure 4:**
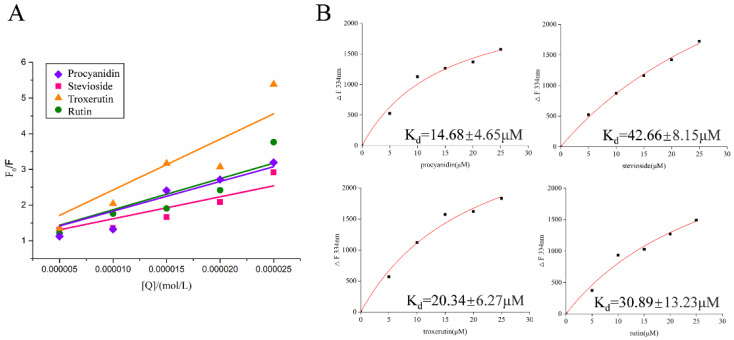
Fluorescence quenching type for the interaction between different compounds and LolB. (**A**): Stern–Volmer plots. (**B**): Plots of compound concentrations vs. changes in fluorescence intensity using One Site-Specific binding model.

To explore the interaction mechanism between the inhibitors and the LolB protein, the Stern–Volmer equation was used to determine the type of fluorescence quenching. Fluorescence quenching can be dynamic, resulting from collisional encounters between the fluorophore and quencher, or static, resulting from the formation of a ground-state complex between the fluorophore and quencher [20]. Figure 4A shows the Stern–Volmer plots of the interactions between the different compounds and LolB, which presented a good linear relationship. The K_q_ values of procyanidin, stevioside, troxerutin and rutin inhibiting LolB were 8.3054 × 10^12^ L/mol·s, 6.1570 × 10^12^ L/mol·s, 14.2317 × 10^12^ L/mol·s and 8.7014 × 10^12^ L/mol·s, respectively (Table 2). These values were much greater than the maximum dynamic quenching rate (2.0 × 10^10^ L/mol·s) [21], confirming that these molecules combined with LolB to form complexes, producing static quenching. According to the One Site-Specific Binding model, the binding constant K_d_ was analyzed to determine the affinity of inhibitors with LolB (Figure 4B). The four K_d_ values were in the micromolar range, especially that of procyanidin, reaching 14.68 ± 4.65 μM, indicative of strong binding effects with LolB protein and stable complex formations.

### 2.5. Analysis of Binding Sites between Active Compounds and LolB

**Figure 5:**
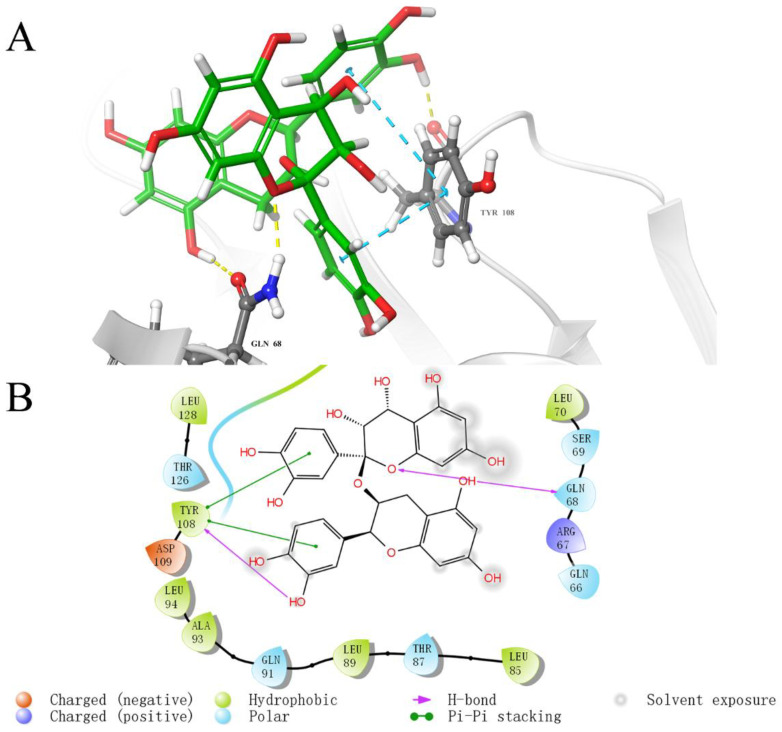
Molecular docking diagram of the interaction between procyanidin and LolB. (**A**): Detailed view of the LolB–procyanidin interaction sites. Amino acid residues involved in binding are shown in stick representation. H bonds are shown as yellow dashed lines and pi–pi stacking interactions are shown as cyan dashed lines. Procyanidin is labeled in green. (**B**): 2D diagram of interaction.

**Figure 6:**
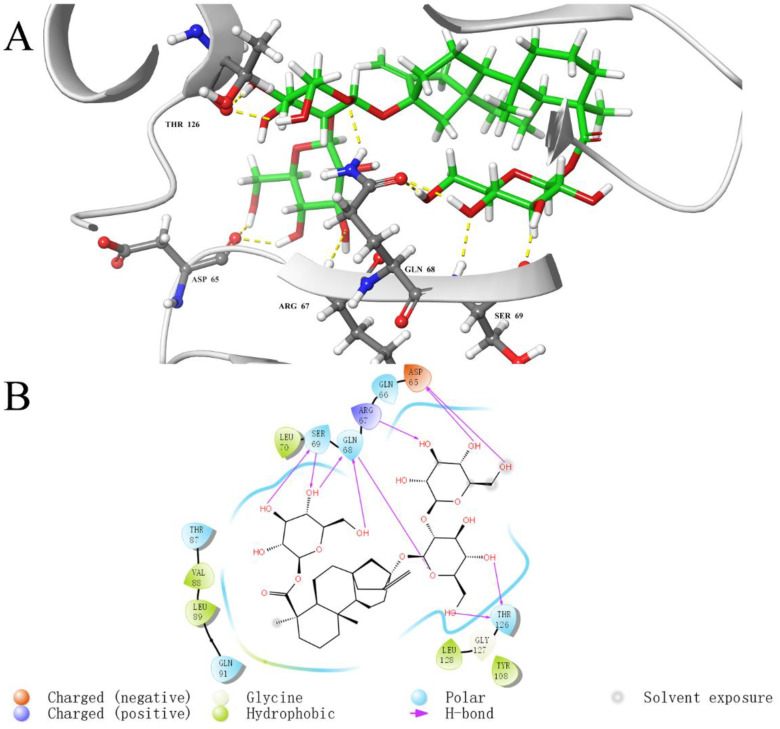
Molecular docking diagram of the interaction between stevioside and LolB. (**A**): Detailed view of the LolB–stevioside interaction sites. Amino acid residues involved in binding are shown in stick representation. H bonds are shown as yellow dashed lines. Stevioside is labeled in green. (**B**): 2D diagram of interaction.

**Figure 7:**
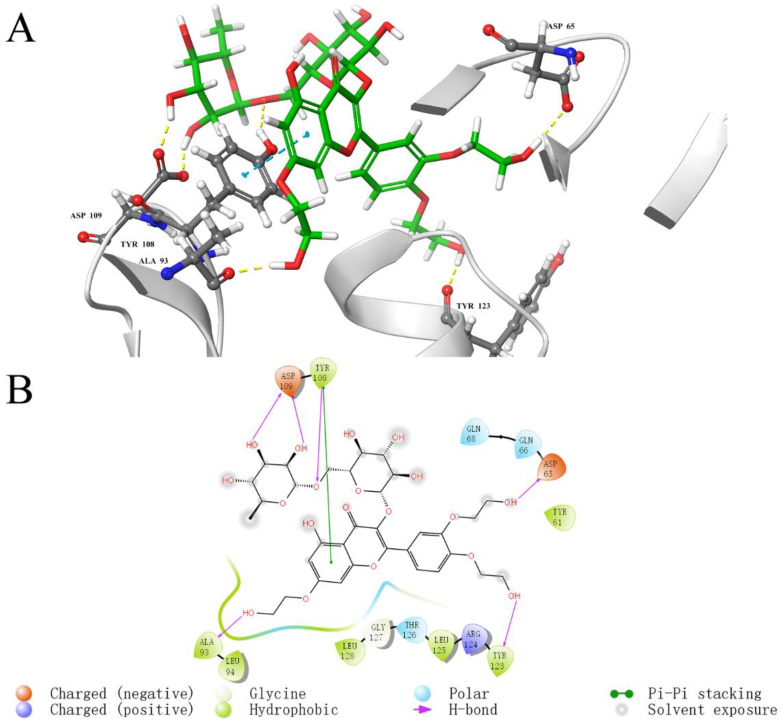
Molecular docking diagram of the interaction between troxerutin and LolB. (**A**): Detailed view of the LolB–troxerutin interaction sites. Amino acid residues involved in binding are shown in stick representation. H bonds are shown as yellow dashed lines and pi–pi stacking interactions are shown as cyan dashed lines. Troxerutin is labeled in green. (**B**): 2D diagram of interaction.

**Figure 8:**
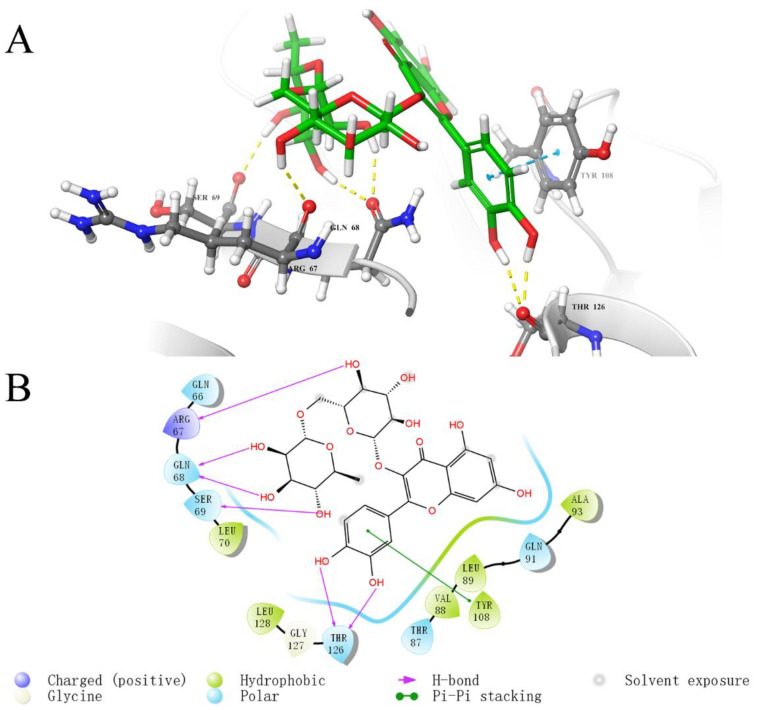
Molecular docking diagram of the interaction between rutin and LolB. (**A**): Detailed view of the LolB–rutin interaction sites. Amino acid residues involved in binding are shown in stick representation. H bonds are shown as yellow dashed lines and pi–pi stacking interactions are shown as cyan dashed lines. Rutin is labeled in green. (**B**): 2D diagram of interaction.

To investigate the key interaction sites between the four natural compounds and the LolB protein, the molecular docking was analyzed through Schrodinger’s Maestro. Figure 5, Figure 6, Figure 7 and Figure 8 describe the interactions between the individual compounds and the LolB protein, showing the docking region and the interaction between ligand and protein. It can be observed, from the 3D diagram of molecular docking, that these natural compounds were bound in the entrance area of the hydrophobic cavity of the critical transport lipoprotein LolB through many main chain contacts. Specifically, the hydroxyl groups and ether bonds of procyanidin formed hydrogen bonds with Tyr108 and Gln68 of the LolB protein, respectively, and the two benzene rings of procyanidin formed pi–pi stacking interactions with Tyr108 (Figure 5). The hydroxyl groups of stevioside formed 10 hydrogen bonds with Thr126, Asp65, Arg67, Gln68 and Ser69 (Figure 6). The hydroxyl groups and ether bond of troxerutin interacted with Tyr123, Asp65, Ala93, Asp109 and Tyr108 of LolB protein (H bonds), and the pi–pi stacking interaction with Tyr108 was also observed (Figure 7). The hydroxyl group of rutin formed hydrogen bonds with Arg67, Gln68, Ser69, Thr126 and Tyr108, and the same pi–pi stacking interaction existed in the complex of rutin and LolB (Figure 8). Consistent with the results of fluorescence spectra (Figure 3), the major acting force, pi–pi stacking interactions and hydrogen bonds, enhanced the hydrophobicity of the LolB protein, then, altered the environment of the amino acids at the entrance of the hydrophobic cavity, affecting the structure and function of OM transport lipoprotein LolB. Given that fluorescence quenching is commonly observed on binding ligands to binding sites, including tryptophan, tyrosine, and phenylalanine [20], the interaction of four inhibitors with LolB might indicate that procyanidin, that formed two pi–pi interactions with Tyr108, had the highest affinity, especially compared to stevioside, the compound with the highest glide score that had no pi–pi stacking.

## 3. Discussion

Nowadays, the AMR of *V. parahaemolyticus* should not be underestimated [22,23]. Since an intact OM is required, both for viability and for resistance against antibiotics, in Gram-negative bacteria, therapeutics targeting OM biogenesis have the potential to kill the bacteria outright and sensitize them to antibiotics that are otherwise unable to penetrate an intact OM [15]. Here, we paid attention to the Lol system, the essential undertaker in transporting OM lipoproteins, and found antibacterial compounds targeting the LolB protein through VS, namely, procyanidin, stevioside, troxerutin and rutin.

**Figure 9:**
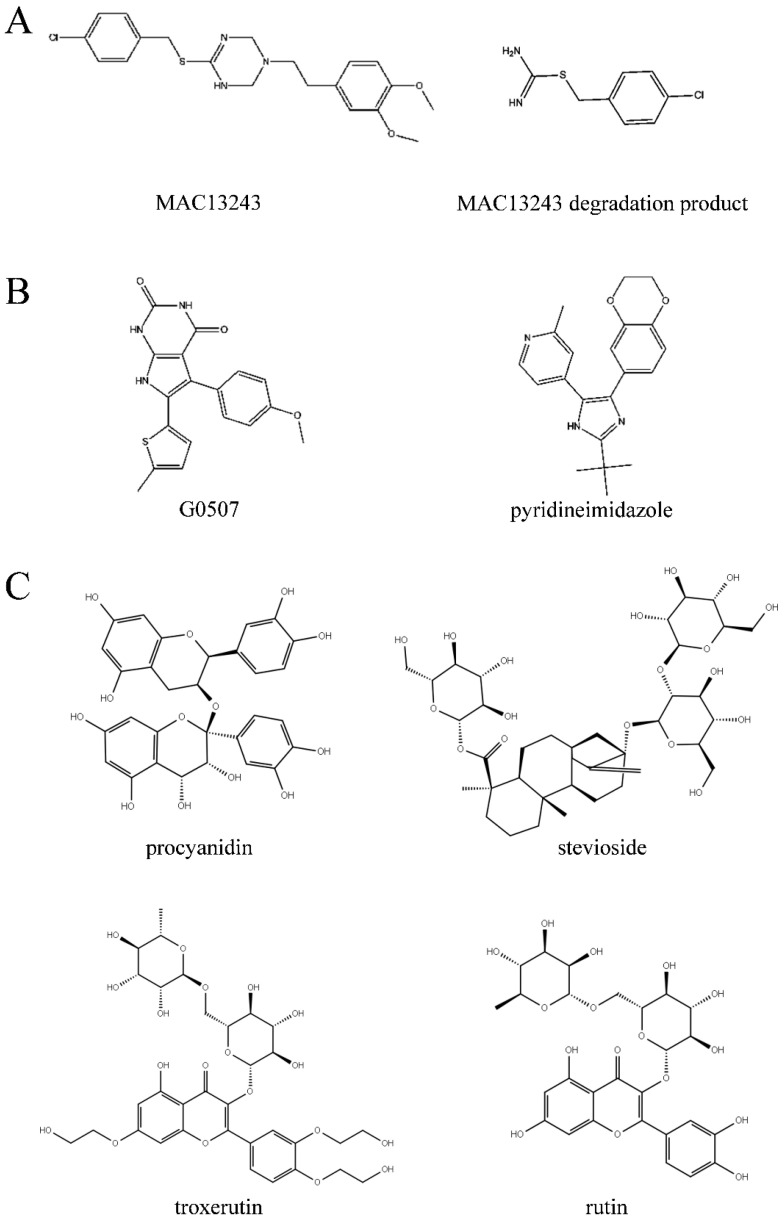
Chemical structures of compounds targeting Lol system. (**A**): LolA inhibitors. (**B**): LolCD_2_E inhibitors. (**C**): LolB inhibitors.

OM biogenesis is involved in specific transport machines, called the Lol, BAM, and Lpt pathways, responsible for transferring lipoproteins, outer membrane proteins and lipopolysaccharide, respectively [24]. The attractiveness of OM biogenesis processes as antibacterial targets is driven, in particular, by their essentiality, conservation, and extra-cytoplasmic localization [25]. In the Lol system, previous research confirmed LolA and LolCD_2_E as antibacterial targets [26]. MAC13243, discovered with cell-based small-molecule screening, had a unique mechanism and promising activity against multidrug-resistant *Pseudomonas aeruginosa* and inhibited the function of the LolA protein (Figure 9) [27]. Moreover, the degradation products of MAC13243 resulted in thiourea compounds, which shared a similar cellular mechanism interacting with LolA (Figure 9) [28]. The first reported inhibitors of the LolCD_2_E complex were the pyridineimidazoles, shown to inhibit the LolA-dependent release of the outer membrane lipoprotein Lpp from *E. coli* spheroplasts [29]. A similar phenomenon was also observed with the presence of the pyrrolopyrimidinedione compound G0507, another LolCD_2_E inhibitor, which was identified in a phenotypic screen for inhibitors of *E. coli* lacking the tripartite efflux pump component, TolC [25]. In this paper, we screened natural inhibitors, procyanidin, stevioside, troxerutin and rutin, in inhibiting *V. parahaemolyticus* (Figure 2), and verified their binding with LolB through in vitro assays (Figure 3), confirming that LolB is a promising drug target. To our knowledge, this is the first demonstration of inhibition of LolB leading to antibacterial activity.

Concerning accessibility, safety and applicability [30], we selected natural compounds as the database, then obtained four LolB inhibitors inhibiting *V. parahaemolyticus* (Table 1 and Figure 2). Firstly, procyanidin is a class of flavonoids from plants like grape seeds [31]. It was demonstrated that procyanidin had clear and well-defined beneficial effects against several pathologies, including cardiovascular heart disease, obesity, where it prevents weight gain and adipose tissue mass increase, and diabetes and insulin resistance [32,33]. Procyanidin also possesses the potency of free radical scavenging, antioxidant activity and cancer chemoprevention [34,35]. Recently, the potential role of procyanidin as a therapeutic agent against SARS-CoV-2 was discovered [35], indicating its promising utility. As the inhibitor of LolB protein with the best glide score in VS (Table 1), stevioside is a natural glycoside extracted from the leaves of *Stevia rebaudiana* [36]. It became well-known for its intense sweetness (250–300 times sweeter than sucrose) and is used as a safe, non-caloric, sweetener in food industries [37]. It is suited for both diabetics, hypertensive and PKU patients, as well as for obese persons intending to lose weight by avoiding sugar supplements in the diet [36]. Troxerutin, also known as vitamin P4, is a naturally occurring flavonoid which is isolated from tea, coffee and cereal grains, as well as from vegetables. It has a variety of valuable pharmacological and therapeutic activities, such as antioxidant, anti-inflammatory, anti-diabetic, anti-tumor, antihyperlipidemic, and nephroprotective activities [38,39]. Furthermore, clinical trials revealed the efficacy of troxerutin for management of phlebocholosis and hemorrhoidal diseases [39]. The last active compound, rutin, also known as vitamin P, or rutoside, is one of the most common dietary polyphenols found in vegetables, fruits, and other plants. It is metabolized by the mammalian gut microbiota and absorbed from the intestines, and becomes bioavailable in the form of conjugated metabolites [40,41]. Rutin showed a wide range of pharmacological applications, including antimicrobial, antifungal, anti-inflammatory, anticancer, antidiabetic, anti-hypertension and anti-hypercholesterolemia, as well as potential antiviral activity against SARS-CoV-2, due to its significant antioxidant and nontoxic properties [42,43]. Taking its numerous benefits into account, rutin can be widely used in functional foods, dietary supplements, and pharmaceuticals. The weaknesses of low aqueous solubility, poor stability and limited membrane permeability need to be resolved in practical application [41,44]. Overall, procyanidin, stevioside, troxerutin and rutin are natural products with low toxicity and have a broad spectrum of pharmacological and therapeutic benefits, compared with synthetic compounds, exhibiting tremendous prospects for development. Admittedly, however, the four compounds are moderate antibacterial inhibitors, which need to be in relatively high concentrations (100 ppm) for inhibition of *V. parahaemolyticus*, and it is possible that they poorly penetrate the OM of Gram-negative bacteria. Future work will focus on enhancing their inhibition effects on *V. parahaemolyticus* and other Gram-negative bacteria, by means of structural optimization.

**Figure 10:**
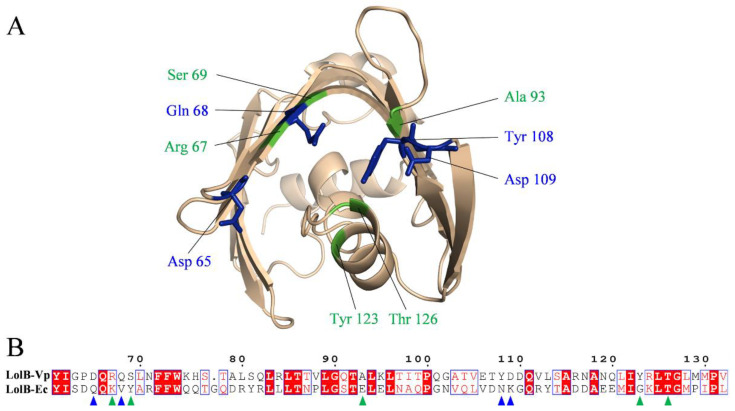
Amino acid residues interacting with inhibitors in *V. parahaemolyticus* LolB. (**A**): Position of interaction sites in *V. parahaemolyticus* LolB. Residues involving backbone contacts are labeled in green, and residues involving side chain interactions are shown as blue sticks. (**B**): Sequence alignment of LolB. Residues involving backbone contacts in *V. parahaemolyticus* LolB are indicated by green triangles, and residues involving side chain interactions in *V. parahaemolyticus* LolB are indicated by blue triangles. Vp, *Vibrio parahaemolyticus*; Ec, *Escherichia coli*.

When resolving the *E. coli* LolB structure, researchers obtained the crystal structure of the complex of LolB with PEGMME2000 at the hydrophobic cavity (PDB 1IWN) [19]. In our study, molecular docking showed the complex of *V. parahaemolyticus* LolB with inhibitor at the entrance of the hydrophobic cavity (Figure 10A and Figure S2), where LolB interacted with LolA in the putative mouth-to-mouth lipoprotein transfer model [45]. Upon binding, the “mouth” of LolB is blocked by the inhibitor, the acyl chain of the lipoprotein is, therefore, unable to enter the hydrophobic cavity of LolB, and the trafficking of lipoprotein and the growth of bacterial cells is possibly prevented. The docking results suggested that the four compounds interacted with LolB through both main chain contacts and side chain contacts (Figure 10A). Some active sites involving backbone contacts, Arg67, Ser69 and Ala93, were located in β-sheet, and Tyr123 and Thr126 positions at the α-helix, that is speculated to undergo conformational changes to give access of the lipoprotein substance to the LolB hydrophobic cavity (Figure 10A) [46]. Therefore, the interaction of stevioside, troxerutin and rutin with Tyr123 and Thr126 likely hindered conformational changes of the α-helix, locked the close state of LolB, and affected LolB functioning. Furthermore, given that the LolB protein has no large structural difference among various microorganisms (Figure 1B), it is quite possible that these backbone contacts also exist between inhibitors and other LolB proteins from different bacteria.

Through analyzing the side chain interactions of the LolB protein with inhibitors, it could be concluded that the residue Tyr108 of *V. parahaemolyticus* LolB was one of the most important active sites, of which the benzene ring and phenolic hydroxyl group of the side chain played a key role for the interaction of procyanidin, troxerutin and rutin with receptor LolB (Figure 5, Figure 7 and Figure 8). Additionally, Gln68 was another important amino acid residue in the *V. parahaemolyticus* LolB protein, since the carboxamido group of Gln68 formed at least two hydroxyl bonds with procyanidin, stevioside, and rutin (Figure 5, Figure 6 and Figure 8). Sequence alignment revealed that the corresponding amino acids of Gln68 and Tyr108 were Val and Asn, respectively, in *E. coli* LolB (Figure 10B). The two residues were specific to *V. parahaemolyticus*, perhaps to procyanidin, interacting with the side chains of Tyr108 and Gln68 only, and could not bind *E. coli* LolB, and, thus, had no better effect on other microbial infections. Moreover, the side chain carboxyl groups in Asp65 and Asp84 were also involved in the formation of hydroxyl bonds, but the residues were not homologous with *E. coli* either. (Figure 6, Figure 7 and Figure 10). In summary, inhibitors targeting *V. parahaemolyticus* LolB, in the future, could be designed and optimized, based on the identified critical residues, Tyr108 and Gln68.

We hope this study can provide strong technical support for the accurate control of *V. parahaemolyticus*, and contribute new strategies for in-depth research in the control of microbial safety. This targeted sterilization technology, aimed at the OM, paves the way for resolving AMR, reducing the risk of bacterial infection and safeguarding human health.

## 4. Materials and Methods

### 4.1. Homology Modeling

The sequence of LolB of *V. parahaemolyticus* RIMD 2210633 (protein id: BAC59004.1) from NCBI GenBank [47] was submitted to SWISS-MODEL (https://beta.swissmodel.expasy.org/, accessed on 1 December 2020) to construct the 3D structure of the protein [48,49]. The most homologous sequence was selected as the template for homology modeling. The effectiveness of the obtained model of *V. parahaemolyticus* LolB, constructed by SWISS-MODEL, was defined by the GMQE&QMEAN method (https://swissmodel.expasy.org/qmean/, accessed on 1 December 2020) [50]. Meanwhile, the PROCHECK program of SAVES v6.0 (https://saves.mbi.ucla.edu/, accessed on 1 December 2020) was performed to validate the conformational rationality of the LolB protein model.

### 4.2. High-Throughput Virtual Screening

The active site of *V. parahaemolyticus* LolB was determined by homologous protein alignment, or prediction through the sitemap module of the Schrodinger Suite (Schrödinger, Inc., New York, NY, USA). After structure preparation (e.g., structure optimization of protein and ligands, receptor grid generation, etc.), 39,000 molecules from the Cayman/2D structure database were used to screen active compounds binding *V. parahaemolyticus* LolB at the large and deep hydrophobic cavity by structure-based virtual screening (provided by APExBIO Technology LLC, Houston, TX, USA). The top 10 compound glide scores were selected and purchased for further research.

### 4.3. Determination of Inhibition on V. parahaemolyticus

*V. parahaemolyticus* RIMD 2210633 strain was a gift from Dr. Craig and Dr. David of the Centre for Environment, Fisheries and Aquaculture Science. The antibacterial effect of the screened compounds was determined by broth microdilution method [51]. Briefly, a single colony of *V. parahaemolyticus*, on Thiosulfate citrate bile salts sucrose (TCBS) agar (Beijing Land Bridge Technology Co., Ltd., Beijing, China), was selected to inoculate into Tryptic Soy Broth (TSB) medium (Beijing Land Bridge Technology Co., Ltd., Beijing, China) containing 3% NaCl, overnight at 37 °C with shaking. The cells were centrifuged at 3000 rpm for 10 min and resuspended in Mueller–Hinton Broth (MHB) medium (Beijing Land Bridge Technology Co., Ltd., Beijing, China), 20 μL of which was dispensed into 96-well plates containing serially diluted compounds (purchased from Shanghai Macklin Biochemical Co., Ltd., Topscience Co., Ltd., Shanghai Acmec Biochemical Co., Ltd., or Shanghai yuanye Bio-Technology Co., Ltd., Shanghai, China) ranging from 100–10 ppm. Polymyxin B was used as control. Plates were incubated statically at 37 °C for 16 h. The OD_600_ of each well was read on Synergy2 Microplate Reader (Agilent Technologies, Inc., Santa Clara, CA, USA). Experiments were repeated three times in triplicate. The inhibition rate of each compound was calculated using the following equation [52]:

Inhibition rate (%) = [1 − (OD_treated_ − OD_compound_)/(OD_control_ − OD_blank_)] ×100% (1)

where OD_blank_ is the OD value of medium before incubation, OD_compound_ is the OD value of medium with compound before incubation, OD_contro_l is the OD value of inoculum and OD_treated_ is the OD value of inoculum with compound.

### 4.4. Cloning, Expression and Purification of V. parahaemolyticus LolB Protein

#### 4.4.1. Cloning of *V. parahaemolyticus* LolB

The DNA sequence of LolB from *V. parahaemolyticus* RIMD 2210633 was obtained from NCBI GenBank [47]. Residues 26-212 of LolB were codon optimized using *E. coli* Codon Usage Analyzer 2.1 (http://faculty.ucr.edu/~mmaduro/codonusage/usage.htm, accessed on 1 September 2020) [53]. A single plasmid, pET28b, containing the codon-optimized gene, was chemically synthesized (GENEWIZ, Suzhou, China) with an N-terminal His_6_ tag and NdeI and HindIII restriction sites.

#### 4.4.2. Expression and Purification of LolB Protein

The LolB protein was acquired through transformation of the expression vector into *E. coli* BL21 (DE3) CodonPlus cells (TIANGEN Biotech Co., Ltd., Beijing, China) grown in LB broth (Beijing Land Bridge Technology Co., Ltd., Beijing, China) in the presence of 50 μg/mL kanamycin at 37 °C. The cells were grown to an OD_600_ of 0.55–0.6, then induced with 0.2 mM isopropyl β-D-1-thiogalactopyranoside (IPTG) for 18 h at 20 °C with shaking at 200 rpm. Cells were harvested, resuspended in buffer A (20 mM Tris, 500 mM NaCl, 10 mM imidazole, 10% glycerol, pH 7.5) containing lysozyme, Complete EDTA-free protease inhibitor (Roche Ltd., Basel, Switzerland) and DNase (Shanghai Macklin Biochemical Co., Ltd., Shanghai, China) and lysed with an Ultrasonic Cell Disruptor (SCIENTZ-IID; Ningbo Scientz Biotechnology Co., Ltd., Zhejiang, China). The insoluble cell lysate was removed by centrifugation at 11,000 rpm for 10 min and the resulting supernatant was loaded onto a 5 mL HisTrap HP column (Danaher Corporation, Washington, DC, USA), washed with buffer B (20 mM Tris, 1000 mM NaCl, 30 mM imidazole, 10% glycerol, pH 7.5) and eluted with buffer C (20 mM Tris, 500 mM NaCl, 300 mM imidazole, 10% glycerol, pH 7.5). Finally, the eluted fractions were purified on a Hiload 16/600 Superdex 200 pg gel filtration column (Danaher Corporation, Washington DC, United States) that was pre-equilibrated with buffer GF (20 mM Tris, 200 mM NaCl, 5% glycerol, pH 7.5).

### 4.5. Fluorescence Spectroscopy

Samples of purified LolB protein were mixed with 0 μM, 5 μM, 10 μM, 15 μM, 20 μM and 25 μM specific compounds dissolved in Tris-HCl buffer, respectively. After 30 min, the fluorescence spectra of the mixtures were measured with a F-7100 fluorescence spectrophotometer (Hitachi High-Tech Corporation, Tokyo, Japan). The excitation wavelength was set at 280 nm, the range of 300–400 nm was selected for emission scanning, and the width of the excitation slit and the emission slit was 5 nm.

### 4.6. Identification of Fluorescence Quenching Mechanism

Based on the data of fluorescence spectra, the type of fluorescence quenching of reaction between compounds and LolB protein was identified by the Stern–Volmer equation [54]:

F_0_/F = 1 + K_q_τ_0_[Q] = 1 + K_sv_[Q] (2)

In the equation, F_0_ and F denote the fluorescence intensities before and after the addition of the compound, respectively. K_q_ and K_sv_ are the biomacromolecule quenching constant and the Stern–Volmer quenching constant, respectively. The value τ_0_ is the average lifetime of the biomacromolecule without the quencher (10^−8^ s) and [Q] represents the concentration of the compound.

The equilibrium binding constant K_d_ was calculated using nonlinear regression with ‘One Site- Specific Binding’ model [55]:

Y = B_max_ * X/(K_d_ + X) (3)

In the equation, X is the ligand concentration, Y is the fluorescence intensity, B_max_ denotes the maximum specific binding.

### 4.7. Analysis of Molecular Docking

The docking results of the top 10 compounds on the *V. parahaemolyticus* LolB protein were visualized by Maestro (Schrödinger, CA, USA) [56]. Ligand–receptor protein interactions were shown in both two-dimensional and three-dimensional plots. In order to clarify the inhibitory mechanism, specific compounds were docked with LolB protein and the distribution of binding sites were analyzed, respectively.

## 5. Conclusions

The development of new antibacterial compounds targeting the Lol system of *V. parahaemolyticus* helps to construct efficient sterilization technology, and could potentially solve the problem of AMR. The present study was carried out to discover active compounds targeting the LolB protein to kill *V. parahaemolyticus*. The present data indicated that procyanidin, stevioside, troxerutin and rutin screened via VS had both exciting binding affinity with LolB and preferable antibacterial activity towards *V. parahaemolyticus* at concentrations of 100 ppm. Fluorescence spectroscopy and molecular docking demonstrated that these active compounds formed stable complexes with LolB through hydrogen bonds and pi–pi stacking interactions, and Tyr108 and Gln68 were the critical active sites in the LolB protein. Our study corroborated LolB as being a promising drug target, elucidated the active sites in LolB, and provided natural antibacterial agents for the targeted elimination of *V. parahaemolyticus*.

## Figures and Tables

**Table 1:** The top 10 compounds targeting LolB obtained from virtual screening.

Number	Glide Score	Compound	Formula	Mol wt
1	−12.023	Stevioside (hydrate)	C_38_H_62_O_19_	822.895
2	−9.858	Forsythoside B	C_34_H_44_O_19_	756.707
3	−9.907	Coenzyme A (sodium salt hydrate)	C_21_H_37_N_7_NaO_17_P_3_S	807.53
4	−8.994	Rutin (hydrate)	C_27_H_36_O_19_	664.566
5	−8.789	Madecassoside	C_48_H_78_O_20_	975.132
6	−8.626	Troxerutin	C_33_H_42_O_19_	742.68
7	−8.307	Procyanidin	C_30_H_26_O_13_	594.525
8	−8.155	Forsythoside A	C_29_H_36_O_15_	624.592
9	−7.847	Mulberroside A	C_26_H_32_O_14_	568.528
10	−7.781	Uridine-5’-diphosphoglucuronic Acid	C_15_H_19_N_2_Na_3_O_18_P_2_	646.23

**Table 2:** Fluorescence quenching constants of the interaction between active compounds and LolB.

Compound	K_sv_ (10^4^ L/mol)	K_q_ (10^12^ L/mol·s)	R^2^
Procyanidin	8.3054	8.3054	0.9817
Stevioside (hydrate)	6.1570	6.1570	0.9802
Troxerutin	14.2317	14.2317	0.9641
Rutin (hydrate)	8.7014	8.7014	0.9700

## Data Availability

Not applicable.

## Supplementary Materials

The following supporting information can be downloaded at: https://www.mdpi.com/article/10.3390/ijms232214352/s1, Figure S1: Ramachandran Plot of *V. parahaemolyticus* LolB model. Figure S2: Virtual screening results containing 70 hits.
