# Supplementary material for: Natural Inhibitors Targeting the Localization of Lipoprotein System in Vibrio parahaemolyticus
Source: Int J Mol Sci. 2022 Nov 18;23(22):14352. doi: 10.3390/ijms232214352 (PMC9696335; doi:10.3390/ijms232214352)
Supplement: Supplementary file 1 [file ijms-23-14352-s001.zip › ijms-1998288-supplementary.pdf]

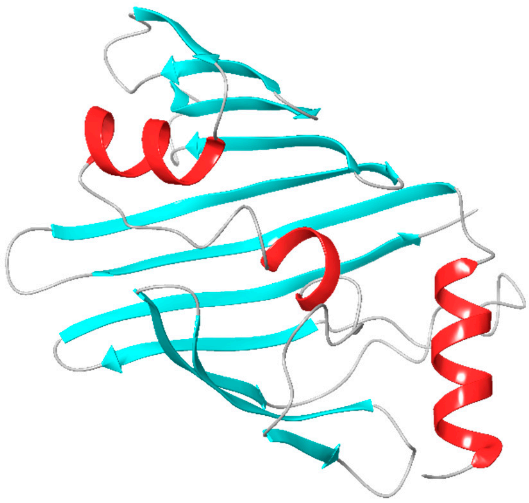

Figure S1. Homology model of *V. parahaemolyticus* LolB protein. α-helixes and β-strands are shown as red and blue.

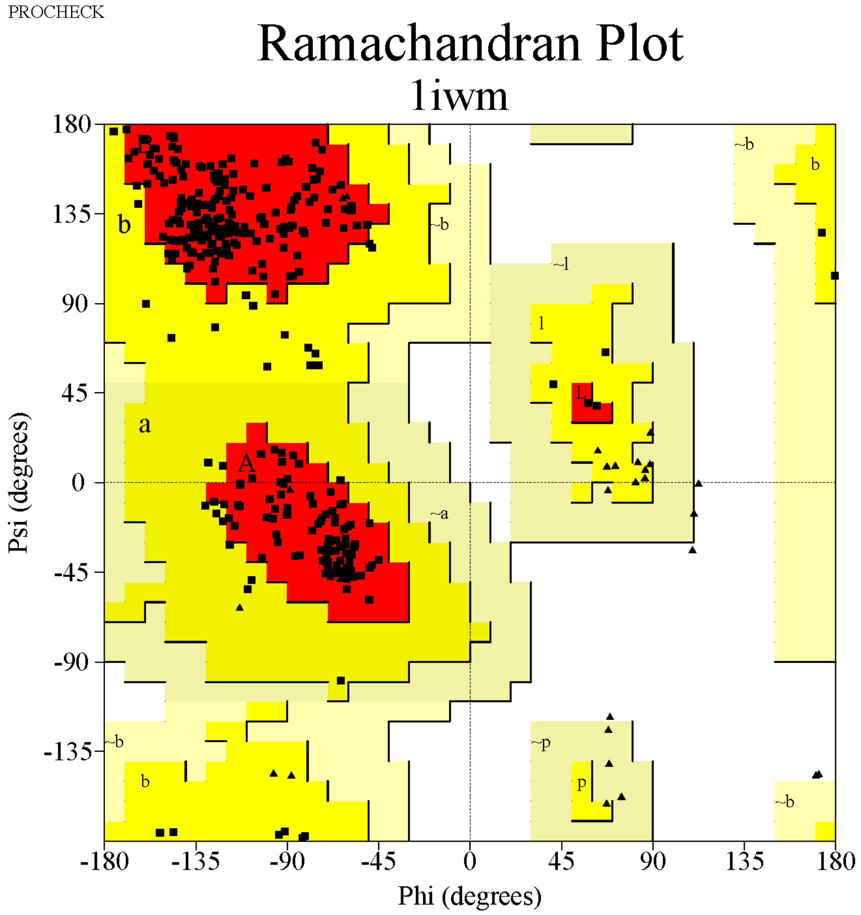

Plot statistics

Residues in most favoured regions [A,B,L]	273	89.8%
Residues in additional allowed regions [a,b,l,p]	31	10.2%
Residues in generously allowed regions [~a,~b,~l,~p]	0	0.0%
Residues in disallowed regions	0	0.0%
	----	------
Number of non-glycine and non-proline residues	304	100.0%
Number of end-residues (excl. Gly and Pro)	2	
Number of glycine residues (shown as triangles)	30	
Number of proline residues	18	

Total number of residues	354	

Based on an analysis of 118 structures of resolution of at least 2.0 Angstroms
and R-factor no greater than 20%, a good quality model would be expected
to have over 90% in the most favoured regions.

1iwm_01.ps

Figure S2. Ramachandran Plot of *V. parahaemolyticus* LolB model.
